# Feeding practices among children attending child welfare clinics in Ragama MOH area: a descriptive cross-sectional study

**DOI:** 10.1186/1746-4358-6-18

**Published:** 2011-11-21

**Authors:** Priyantha J Perera, Meranthi Fernando, Tania Warnakulasuria, Nayomi Ranathunga

**Affiliations:** 1 Department of Paediatrics, Faculty of Medicine, University of Kelaniya, Sri Lanka and North Colombo Teaching Hospital Ragama, Sri Lanka; 2 Department of Paediatrics, Faculty of Medicine, University of Kelaniya, Sri Lanka; 3 Clinical skills laboratory, Faculty of Medicine, University of Kelaniya, Sri Lanka

## Abstract

**Background:**

Feeding during early childhood is important for normal physical and mental growth as well as for health in later life. Currently, Sri Lanka has adopted the WHO recommendation of exclusive breastfeeding for six months, followed by addition of complementary feeds thereafter, with continuation of breastfeeding up to or beyond two years. This study was conducted to evaluate the current feeding practices among Sri Lankan children during early childhood.

**Methods:**

This study was a descriptive cross-sectional study conducted in the Ragama Medical Officer of Health (MOH) area. It was conducted between 10 August 2010 and 30 October 2010. Children between the ages of 24 and 60 months, attending child welfare clinics, were included in the study on consecutive basis. An interviewer-administered questionnaire was used to collect data regarding sociodemographic characteristics and feeding practices.

**Results:**

There were 208 boys and 202 girls in the study population. Of them, 255 (62.2%) were exclusively breastfed up to 6 months. Younger children had a statistically significant, higher rate of exclusive breastfeeding compared to older children. Three hundred and fifty one (85.6%) children had received infant formula, and it was started before the age of 6 months in 61 children, and in 212 before one year. Sugar was added to infant formula in 330 (80.4%) children, and out of them 144 had sugar added within first year of life. Complementary foods were started before 4 months in 29 (7%) children. Of the 410 children, 294 (71.7%) were breastfed beyond 2 years and 41.6% of them were breastfed at regular intervals throughout the day. Three hundred and thirty eight (82.6%) children were receiving overnight feeding of either breast milk or infant formula even after 2 years.

**Conclusions:**

Though a high rate of exclusive breastfeeding was observed in this study population, there are many other issues related to feeding during the early years of life that need immediate intervention. Too early introduction of complementary food, using infant formula without an indication, adding sugar to infant formula, too frequent breastfeeding and overnight feeding of older children are among them.

## Background

Correct feeding practices from early childhood are important for normal physical and mental growth, to have normal development, optimum immunity, reduce atopic conditions and training the child in correct feeding habits. It is well established that under-nutrition as well as obesity in early childhood is associated with increased morbidity and mortality in later life [1]. With a view of optimizing nutrition among children, in 2001 WHO recommended that infants be exclusively breastfed for the first six months and thereafter to be given nutritious complementary food and continue breastfeeding up to the age of two years or beyond [2]. WHO defines exclusive breastfeeding as giving an infant only breast milk, excluding solids or any other fluids (including infant formula) except medicines, vitamins, and minerals [3].

Sri Lanka adopted this recommendation in 2005 and up to that time the practice was to continue exclusive breastfeeding up to 4-6 months. According to Sri Lanka Demographic and Health Survey (DHS) 2006/07, by the Department of Census and Statistics, published in 2009, 75.8% children between 0 and 5 months received exclusive breastfeeding, which is highest for the South East Asian region [4]. This figure is based on 24 hour recall method, where a mother is questioned regarding food that was given to her child during previous 24 hour period. This is the method recommended by WHO to assess the exclusive breastfeeding rate. The major limitation of this method is that it does not consider what food was given to the child previously and thereafter. A study conducted in Kalutara district in Sri Lanka by Agampodi et al has shown that the actual exclusive breastfeeding rate is lower than the figures obtained by 24 hour recall [5].

Presently, Sri Lanka is considered as a moderate income country and has impressive health statistics, such as a maternal mortality rate of 33.4 per 100,000[6] and infant mortality rate of 10.1 per 1000 live births [7]. Sri Lanka is divided in to 25 administrative districts and Ragama is situated in Gampaha district which is the second most populous district in Sri Lanka. Gampaha district had 9.2% of poor households in 2002 and only Colombo district had a better figure [8]. Ragama is situated about 25 kilometers north of the capital Colombo, and mainly consists of urban and suburban areas. The geographical extent of the Ragama MOH area is about 25 km^2 ^and consists of a socio-economically mixed population. It had an estimated population of 75,591 and 15,137 housing units in 2005. A child welfare clinic is held twice a week within the MOH area, which is conducted by the doctors of the MOH office and public health midwives. Children under 5 years of age are brought to these clinics for immunization, nutritional assessment, health education, growth monitoring and clinical examination. According to MOH office statistics, about 95% of the children living in the area are followed up in these clinics.

Though the breastfeeding rate in Sri Lanka is reported to be high, in our clinical observations we often come across many inappropriate feeding practices during early childhood. Therefore, this study was conducted in Ragama Medical Officer of Health (MOH) area, with the objective of assessing feeding practices among Sri Lankan children during early childhood.

## Methods

The study was a descriptive cross-sectional study conducted between 10 August 2010 and 30 October 2010, at child welfare clinics held in Ragama MOH area. The reason for selecting Ragama MOH area is because it consists of a socio economically mixed population and easy accessibility for the investigators.

Four hundred and ten children between 24 and 60 months of age attending child welfare clinics were included in the study. Permission to conduct the study was obtained from Medical Officer of Health, Ragama and informed written consent was obtained from mothers to include their children in the study. All children in the above age category who attended the clinics with their mothers during the study period were recruited on a consecutive basis. None of the mothers refused to give consent.

As the policy of exclusive breastfeeding for 6 months was introduced to Sri Lanka in 2005, a child who was born in this year would be 5 years by 2010. This was the rationale for recruiting children up to 60 months of age for the study. As the objective of the study was to assess feeding practices during early childhood such as; exclusive breastfeeding, introduction of complementary foods, use of infant formula, frequency of breastfeeding, overnight feeding and adding sugar to infant formula, children above 2 years of age needed to be included in the sample. Therefore, children between 24 months and 60 months of age were recruited for the study. According to the DHS survey, the exclusive breastfeeding rate up to 6 months is around 76% and from our clinical observations about 70% of mothers were continuing breastfeeding beyond 2 years. A sample size of 323 is required to estimate this to within 5% (prevalence 70%, 95 confidence interval 65% to 75%). Preliminary inquiries revealed that during a month, about 125 children in the age group of 2 to 5 years attend child welfare clinics in the area. Therefore, children attending child welfare clinics within a 3 month period were recruited for the study.

The study instrument consisted of an interviewer-administered questionnaire, which was pretested at the clinics of Ragama Teaching Hospital and validated for this purpose. Children visiting for services from Ragama Teaching Hospital, but living outside Ragama MOH area were used for pretesting, to avoid the same children being included in the study. The questionnaire consisted of two parts. Part one was used to get demographic characteristics of the mothers. Part two was used to record information regarding feeding practices. Data collection was done by two pre-intern doctors who are the second and third authors of this manuscript. The data collection process was supervised by the first author who is a consultant paediatrician.

Mothers were questioned about how they fed their children during the first five years. Mothers were allowed to describe how they fed their children and specific questions were asked either to verify the validity of the information, or help the mother to remember details. Questions like whether mothers gave any food or water other than breast milk during first 6 months were specifically asked. In Sri Lanka, the third dose of DPT + oral polio vaccine is given at 6 months, so mothers were asked whether they were exclusively breastfeeding their children by the time of third dose of these vaccines. Almost all mothers did not have difficulty in remembering the details about feeding. Before starting data collection, agreements were made regarding what questions to be asked during the interview and how the questions would be explained to mothers, to minimize the inter-observer bias. To avoid the same child being included more than once in the study, mothers were questioned whether they had been interviewed before.

Exclusive breastfeeding was defined as feeding the child only with breast milk, without giving solids or any other food (not even water) for a specified period since birth, but giving vitamins, medicines, minerals and oral rehydration solution was allowed. Age of starting complementary feeding was defined as the age at which solids and semi-solids were introduced to the child in addition to breast milk. Overnight feeding was defined as a child continuing to feed while sleeping during the night, without rinsing the mouth with water after the feed. Age of the child was defined as child's age in completed months by the date of data collection. To ascertain whether a child was frequently breastfeed after two years of age, the mother was questioned whether her child was breastfed several times a day when the child was requesting it, without any particular timing or pattern, and whether breastfeeding was offered when the child refused a main meal. Duration of breastfeeding, and age at which other nutritional interventions were done, were recorded as the age in completed months.

The research proposal was reviewed by the research and ethics committee of Sri Lanka College of Paediatricians and ethical clearance was granted.

The data was analyzed using SSPS version 16 and Chi square for trend was used to test statistical significance.

## Results

Four hundred and ten children between 24 months and 60 months of age were included in the study. There were 208 boys and 202 girls.

The majority of the mothers in the study population had a satisfactory level of education and only one mother (0.2%) had never gone to school. Mean monthly income of a family was Rs 22,580.49. Sociodemographic characteristics of the study population are given in Table 1.

**Table 1:** Sociodemographic characteristics of the study population

Characteristics	Frequency (n = 410)	Percentage (%)
**Sex**		
Male	208	50.7
Female	202	49.3
Age*		
2 years(24-35 months)	125	30.5
3 years(36-47 months)	154	37.6
4 years(48-60 months)	131	31.9
**Siblings**		
Have siblings	207	50.5
No siblings	203	49.5
**Mother's education**		
None	1	0.3
Up to grade 5	5	1.2
Grade 5 to 11	247	60.3
Grade 12 to 13	140	34.1
University	17	4.1
**Income(Rs.)†**		
< 15000	111	27.1
15000-24999	148	36.1
> = 25000	151	36.8

* Age was defined in completed months by the date of data collection.

† According to current rates, US$1 equal to Rs.110

### Feeding pattern

Two hundred and fifty five (62.2%) children were exclusively breastfed until the completion of 6 months, while 1% were never exclusively breastfed, and 4.4% continued exclusive breastfeeding beyond 6 months. Table 2 gives the distribution of the sample according to the duration of exclusive breastfeeding.

**Table 2:** Duration of exclusive breastfeeding

Age in months	Frequency (n = 410)	Percentage (%)
0	4	1.0
1	2	.5
2	6	1.5
3	22	5.4
4	59	14.3
5	44	10.7
6	255	62.2
7	11	2.7
8	7	1.7
Total	410	100.0

Distribution of number of children exclusively breastfed for 6 months, according to their age at the time of data collection, is depicted in Table 3. The exclusive breastfeeding rate has increased to 79.2% from 50.4% over a period of three years since the introduction of new recommendations. The increase that was observed in exclusive breastfeeding rates with time was statistically significant when Chi-square for trend was applied (p < 0.001).

**Table 3:** Trend in exclusive breastfeeding rate according present age of the child

	Exclusively breastfed		
		
Present age in months	Up to 6 months (%)	< 6 months (%)	Odds Ratio (95% CI)
24-35 (n = 125)	99 (79.2)	26 (20.8)	3.75 (2.09, 6.79)
36-47 (n = 154)	108 (70.1)	46 (29.9)	2.31 (1.38, 3.87)
48-60 (n = 131)	66 (50.4)	65 (49.6)	1.00

Chi-square for trend = 24.037 DF = 1 p < 0.001

Of 410 children, 351 (85.5%) had received infant formula at some time in their life. When formula feeds were given 192 (54.7%) used a cup, while 150 (42.7%) used a bottle and teat for feeding. Both cup and bottle were used by 8 (2.3%) and 1 (0.3%) used a spoon. Infant formula was introduced at different ages, and 330 children had sugar added to their milk. The age at which infant formula was commenced, and age at which sugar was added for the first time to formula, is demonstrated in Table 4.

**Table 4:** Age when formula feeding commenced and sugar was first added to infant formula

Age	Frequency (n = 351)	Percentage (%)
**Age at which formula feeding started**		

0-6 months	61	17.4
7-12 months	151	43.0
13-18 months	52	14.8
19-24 months	46	13.1
25-30 months	14	4
31-36 months	25	7.1
> 36 months	2	0.6

**Age at which sugar added to infant formula**		

Not added sugar	21	6.0
0-6 months	12	3.4
7-12 months	132	37.6
13-18 months	58	16.5
19-24 months	61	17.4
25-30 months	19	5.4
31-36 months	39	11.1
> 36 months	9	2.6

As per information given in Table 5, 257 (62.7%) children had complementary foods started at 6 months. However, 6.4% of children had complementary foods commenced before 4 months, and 4.4% had it delayed beyond 6 months.

**Table 5:** Age at which complementary feeding started

Age in months	Frequency (n = 410)	Percentage (%)
2	4	1.0
3	22	5.4
4	64	15.6
5	44	10.7
6	257	62.7
7	11	2.6
8	8	2.0

Breastfeeding was continued beyond the age of 2 years for 294 (71.7%) children and of them, 30.6% were breastfed only at night, but others were breastfed on demand throughout the day. Overnight feeding was received by 338 (82.6%) children, even after the age of 2 years. Of them, 65 were on infant formula, 200 on breast milk and 73 on both.

## Discussion

Before current WHO recommendations came in to effect in 2005, the recommendation in Sri Lanka was to continue exclusive breastfeeding up to 4-6 months. In this study population, 67% of children had received exclusive breastfeeding up to 6 months. Children included in this study were between 24 and 60 months by the date of data collection in 2010. Some children included in the study were born in the same year in which the recommendation on duration of exclusive breastfeeding was changed. When a new recommendation is implemented it takes some time for it to get established. This study clearly demonstrates how exclusive breastfeeding rates up to 6 months have improved with time since implementation of the new recommendation in 2005. The exclusive breastfeeding rate up to 6 months in children who were 24 to 35 months of age at the time of the study was 79%, which is very high compared to other countries in the region.

Ministry of Health of Sri Lanka has enforced a strict breastfeeding code which strongly supports exclusive breastfeeding [9]. Ministry of Health has also issued infant and young child feeding guidelines, which give specific instructions regarding exclusive breastfeeding and feeding after six months [10]. In addition, most of the 10 steps in baby friendly hospital initiative, which are aimed at initiating and promoting exclusive breastfeeding at the time of birth, are adopted by hospitals in Sri Lanka. These factors, plus hard work done by primary health care workers in Sri Lanka, would have contributed to the high exclusive breastfeeding rates observed. An interventional study has been conducted in Gampaha district in 2006 on breastfeeding, which also trained public health midwives in lactation management [11]. This also would have contributed to the above results.

The present economic state in the country demands females to be employed to meet the ever increasing cost of living. Female labour force participation in Sri Lanka was 32.8% in 2009 [12]. In districts like Badulla, Nuwaraelliya, and Anuradhapura this was above 40%. Females employed in the government sector are entitled to full pay maternity leave for only 84 working days, excluding weekends and public holidays. This is equivalent to about 4 calendar months in Sri Lanka. Thus a mother planning to breastfeed exclusively for 6 months will have to take half pay leave as well. Further, lactating mothers working in the private sector do not enjoy same privileges as government employees. Therefore, if we are looking forward to improve exclusive breastfeeding rates further, it is important that fully paid maternity leave is extended up to 6 months, and that private sector employers also comply with regulations regarding maternity leave.

Of a total of 410 children, 17% of babies were on formula feeds and 34% were on complementary foods before 6 months of age. There is evidence that the early introduction of solids or formula can increase the risk of allergies and atopic conditions in later life. A review published in 2008 found that the risk of allergies in later life is high if solids were introduced before 3 to 4 months of age [13]. However, the issue of adding solid foods after 4 months is more complex, where some studies have even shown reduced incidence of allergies when solids are introduced after 4 months rather than at 6 months [14]. In this study, 23% of children had received complementary foods by 4 months, and almost all of these mothers had started solids without medical advice. A significant number of children (4%) had the introduction of complementary foods delayed up to 7 months, and in 2% of them it was delayed as much as 8 months. Delayed introduction of complementary feeding is a well known cause of growth failure and iron deficiency, as breast milk alone cannot provide adequate nutrients required by a child after 6 months. These issues regarding complementary feeding need urgent attention and correction.

A surprisingly high percentage (86%) of children have had infant formula, and more than half of these were on infant formula during the first year of life. Some mothers believe it is essential to give infant formula to children. It is not uncommon to find babies of non-working mothers who have adequate breast milk, are also on infant formula. When inquiries are made into the dietary history of these children, it is often revealed that there is no real indication for infant formula. When mothers are employed and away from home for a long period there may be a need to start infant formula for their children. However, when mother is away from home only for a short period of the day, expressed breast milk and complementary foods may be sufficient to support child's nutrition until the mother returns home to breastfeed. There should be clear instructions to mothers regarding when to consider starting infant formula for their children and to seek medical advice before commencing it.

Adding sugar to infant foods is not recommended until one year of age. This is because it will suppress the appetite and reduce the intake of proteins, increase the risk of dental caries, cause obesity and concerns about diabetes mellitus in later life. In this study, about 4% of children had sugar added to infant formula before 6 months, and about 44% had sugar added before one year.

Overnight feeding with infant formula is an identified risk factor for dental caries [15]. Adding sugar to infant formula increases this risk substantially. However, there is no clear evidence in medical literature regarding the association between overnight breastfeeding and dental caries. Continuing overnight feeding for a long duration seems to be a common practice, as 83% of children were receiving overnight feeding even beyond 2 years of age. Though it is mainly with breast milk, 138 children (34%) were receiving overnight feeding with infant formula. Therefore, it is important to educate mothers not to continue overnight feeding in older children and not to add sugar to formula, even if it has to be given due to a specific indication.

Substantial numbers of children (72%) are breastfed beyond 2 years, and 42% of them are breastfed frequently throughout the day. Continuing too frequent breastfeeding, especially on demand, and offering breastfeeding when a child refuses a main meal will result in the child developing lack of interest in solids. This will result in growth faltering, as breast milk alone cannot support the growth of a child beyond 6 months. We very often come across children brought in by their parents complaining that child has poor appetite for solids, only to find that it is due to too frequent breastfeeding. There are specific instructions regarding breastfeeding beyond 2 years given in "Infant and young child feeding guidelines for Sri Lanka" issued by the Ministry of Health. It states that "Breastfeeding should be continued during the second year up to two years or beyond, given after main meals, not to breastfeed before main meals and not to replace a main meal with breast milk". However, these instructions either have not reached mothers or they have ignored them. We feel that correcting breastfeeding practices after 2 years is as important as enforcing exclusive breastfeeding during the first 6 months.

The main limitation of this study is the possible recall bias involved in collecting information regarding feeding practices in retrospect. The bias is likely to be high when a mother has more children. An educated mother is more likely to remember these details accurately, as she will be aware of the importance of this information, and may even keep records of them. In Sri Lanka, total fertility rate was 2.3 in 2006, and the female literacy rate in Gampaha district was 96.6 [16]. Under such circumstances, we can assume that mothers will have a better chance of remembering details about their children. Further, during data collection we helped mothers to remember information by correlating feeding practices to other events like immunization. It was remarkable that almost all mothers recruited for the study had no difficulty in remembering exact details of their babies. However, we acknowledge that the recall bias would not have been totally eliminated.

This study was carried out in an area with close proximity to the capital and in an area with better socioeconomic condition than most parts of the country. Thus, we can assume that the situation in rest of the country is either similar or worse than this. Therefore, an interventional programme aimed at correcting issues highlighted by this study is indicated urgently.

## Conclusions

This study clearly highlights that although the exclusive breastfeeding rate is satisfactory in this study population, there are many inappropriate feeding practices during early childhood. Therefore, reinforcement of instructions stated in "Infant and young child feeding guidelines for Sri Lanka" regarding the introduction of complementary feeds and continuation of breastfeeding beyond two years is indicated.

## Competing interests

The authors declare that they have no competing interests.

## Authors' contributions

PJP was responsible for planning the research, writing the research proposal, writing the manuscript, supervising data collection and data analysis. MF and TW contributed in planning the research and were mainly involved in data collection and analysis. NR contributed in planning the research, analyzing data and writing the manuscript. All authors have gone through the final version of manuscript and accepted it for publication.

## Authors' information

PJP is a board certified consultant paediatrician in Sri Lanka and is the head of department of paediatrics and a senior lecturer in paediatrics attached to Faculty of Medicine, University of Kelaniya, Sri Lanka.

MF and TW, are demonstrators attached to Department of Paediatrics, Faculty of Medicine, University of Kelaniya, Sri Lanka.

NR is a demonstrator attached to the clinical skills lab, Faculty of Medicine, University of Kelaniya, Sri Lanka.
